# Intracellular inflammatory and antioxidant pathways in postmortem frontal cortex of subjects with major depression: effect of antidepressants

**DOI:** 10.1186/s12974-018-1294-2

**Published:** 2018-09-04

**Authors:** David Martín-Hernández, Javier R. Caso, J. Javier Meana, Luis F. Callado, José L. M. Madrigal, Borja García-Bueno, Juan C. Leza

**Affiliations:** 1 Centro de Investigación Biomédica en Red de Salud Mental (CIBERSAM), Instituto de Salud Carlos III (ISCIII), C/ Monforte de Lemos 3-5, 28029 Madrid, Spain; 2 Departamento de Farmacología y Toxicología, Facultad de Medicina, Universidad Complutense de Madrid, Instituto Universitario de Investigación en Neuroquímica UCM, Avda. Complutense s/n, 28040 Madrid, Spain; 3 Instituto de Investigación Sanitaria Hospital 12 de Octubre (Imas12), Avda. de Córdoba, s/n, 28041 Madrid, Spain; 4 Departamento de Farmacología, Universidad del País Vasco, UPV/EHU, B. Sarriena s/n, 48940 Leioa, Bizkaia Spain; 5 Instituto de Investigación Sanitaria Biocruces, Plaza de Cruces s/n, 48903 Barakaldo, Bizkaia Spain

**Keywords:** Major depression, Postmortem frontal cortex, Antidepressants, Neuroinflammation, Toll-like receptor 4 pathway, Mitogen-activated protein kinases, Nrf2 pathway

## Abstract

**Background:**

Studies show that Toll-like receptors (TLRs), members of the innate immune system, might participate in the pathogenesis of the major depressive disorder (MDD). However, evidence of this participation in the brain of patients with MDD has been elusive.

**Methods:**

This work explores whether the protein expression by immunodetection assays (Western blot) of elements of TLR-4 pathways controlling inflammation and the oxidative/nitrosative stress are altered in postmortem dorsolateral prefrontal cortex of subjects with MDD. The potential modulation induced by the antidepressant treatment on these parameters was also assessed. Thirty MDD subjects (15 antidepressant-free and 15 under antidepressant treatment) were matched for gender and age to 30 controls in a paired design.

**Results:**

No significant changes in TLR-4 expression were detected. An increased expression of the TLR-4 endogenous ligand Hsp70 (+ 33%), but not of Hsp60, and the activated forms of mitogen-activated protein kinases (MAPKs) p38 (+ 47%) and JNK (+ 56%) was observed in MDD. Concomitantly, MDD subjects present a 45% decreased expression of DUSP2 (a regulator of MAPKs) and reduced (− 21%) expression of the antioxidant nuclear factor Nrf2. Antidepressant treatment did not modify the changes detected in the group with MDD and actually increased (+ 25%) the expression of p11, a protein linked with the transport of neurotransmitters and depression.

**Conclusion:**

Data indicate an altered TLR-4 immune response in the brain of subjects with MDD. Additional research focused on the mechanisms contributing to the antidepressant-induced TLR-4 pathway modulation is warranted and could help to develop new treatment strategies for MDD.

**Electronic supplementary material:**

The online version of this article (10.1186/s12974-018-1294-2) contains supplementary material, which is available to authorized users.

## Background

Many studies are focusing on identifying new etiopathological trails and therapeutic targets of depression. One promising development is the apperception of the inflammation, both in the brain and periphery, as an element worth to consider in the pathogenesis of major depressive disorder (MDD). This concept is grounded in the detection of activated inflammatory/immune response in this disease [1, 2] both in the brain and in the periphery.

Studies demonstrate that MDD is associated with manifestation of inflammatory markers [3]. Moreover, meta-analyses show increased inflammatory parameters in the blood of patients with MDD [4, 5]. Alternatively, patients undergoing cytokine therapy develop depressive symptoms [6, 7] and experimental-induced inflammation promotes mood deterioration in healthy subjects [8]. Furthermore, elevated inflammatory markers predict a poorer response to antidepressants (AD) while non-responding patients to AD show persistently elevated inflammation [9].

Currently, increasing attention is being paid to the potential role of the innate immune system in the pathogenesis of psychiatric diseases, particularly to Toll-like receptors (TLRs) [10]. TLRs trigger a complex proinflammatory cascade that can regulate central nervous system (CNS) homeostasis and even promote pathology [11]. TLRs are expressed not only in immune cells, but also in neurones, astroglia, and resident microglia [12–14]. Their ubiquitous distribution suggests that TLRs play other roles in non-pathogen-associated CNS diseases/injuries, presumably through recognizing endogenous molecules released from damaged tissues, also known as damage-associated molecular patterns (DAMPs) (e.g., heat shock protein Hsp60, Hsp70) [15].

Most of studies have focused on TLR-4, which responds predominantly to lipopolysaccharide (LPS), a component of the outer membrane of Gram-negative bacteria [16]. TLR-4 triggers a transduction pathway (i.e., specific kinases including mitogen-activated protein kinases —MAPKs) resulting in the activation of inflammatory nuclear transcription factors [17]. Many of the MAPK pathway downstream targets are transcription factors that coordinate the transcription of several genes encoding inflammatory mediators, such as cytokines, chemokines, and inducible pro-oxidative enzymes [17].

Despite the considerable evidence suggesting active inflammatory pathways in MDD, confirmation of abnormalities in the postmortem brain of affected subjects has been in some way elusive. Increased cytokine and chemokine gene expression and/or concentrations have been shown in the frontal cortex of MDD subjects [18–21]. Aditionally, altered microglial kynurenines have been described in the cingulate gyrus and hippocampus of MDD [22, 23] and an astrocytic hypertrophy in the anterior cingulate white matter of depressed suicides has been reported [24]. Moreover, a recent study employing dentate gyrus proposes neuroinflammation as crucial in MDD [25].

An important limitation in brain postmortem studies is the possibility that presence of antidepressant treatment could affect the expression and function of cytokines and other inflammatory factors. Most of the previous postmortem studies were performed under experimental conditions that were not designed to control for this factor.

The present work aims to study whether specific elements of intracellular pathways controlling inflammation along with factors related to the restraining of the oxidative/nitrosative stress (i.e., the nuclear factor Nrf2) are altered in postmortem dorsolateral prefrontal cortex of subjects with MDD. In particular, this study has focused on innate immunity-related elements ordinarily understudied and novel parameters such as MAPKs and the antioxidant nuclear factor Nrf2 pathways, respectively. The potential modulation induced by the antidepressant treatment on these parameters was also assessed.

## Methods

### Postmortem human brain samples

Human brain samples from dorsolateral prefrontal cortex (Brodmann’s area 9) were obtained at autopsy in the Basque Institute of Legal Medicine, Bilbao, Spain, in compliance with research policies and ethical committees for postmortem brain studies. After a retrospective search for *antemortem* medical information, 30 Caucasian subjects diagnosed with MDD (DSM-IV, DSM-IV-R, or CIE-10 criteria) were matched for ethnic origin, gender, and age (± 4 years) to 30 control subjects in a paired design. Since non-compliance is frequently observed, the absence or presence of antidepressant drugs was defined according to the toxicological screening at the time of death. Thus, MDD subjects were divided into two groups: subjects antidepressant-free at time of death (AD-free; *n* = 15) and subjects antidepressant-treated at time of death (AD-treated; *n* = 15), see Additional file 1 for full details about sample and demographic information.

### Preparation of nuclear and cytosolic extracts

A widely utilized method that provides a high purity nuclear fraction, practically without cytosolic contamination [26] was used (see Additional file 1).

### Western blot analysis

The expression levels of TLR-4, Hsp60, Hsp70, phospho-ERK 1/2, phospho-JNK, phospho-p38, p38 α/β, PI3K, Keap-1, and S100A10 (p11) in cytosolic extracts and the expression levels of DUSP-2, Nrf-2, and p65 (NF-κB subunit) in nuclear extracts from brain samples were analyzed through Western blot (see Additional file 1).

### Protein assay

Protein levels were measured using Bradford method based on the principle of protein-dye binding.

### Chemicals and statistical analyses

Unless otherwise stated, the chemicals were from Sigma-Aldrich (Spain).

Estimation of the sample size was drawn from previous postmortem studies of neuroinflammation, apoptosis, and oxidative/nitrosative stress in schizophrenia [27] and MDD [28].

Data are expressed as mean ± SEM. Protein expression data were normalized for respective endogenous control expression (β-actin or GAPDH) and relative to a reference standard (pool of control samples), whose expression level was defined as 100%. The quantification procedure was repeated three times for each pair of subjects in different gels. The mean value of the different gels was used as a final estimate.

Unpaired two-tailed *t* test was performed when comparing controls vs MDD and a two-tailed *t* test with Welch’s correction was employed when samples did not have equal standard deviations. After completion of the assays, since the control groups for AD-free and AD-treated subjects did not differ from each other for confounding factors (see Additional file 1: Table S1), they were pooled for further statistical analyses. One-way ANOVA with a Fisher’s LSD post hoc test was used to compare between controls and AD-free and AD-treated MDD groups. Data were analyzed using the Brown-Forsythe test to assess the Gaussian distribution. In cases in which data did not follow a Gaussian distribution, a Kruskal-Wallis test followed by post hoc Dunn’s test was performed. Correlations were performed to evaluate the influence of the postmortem delay, storage time, and body mass index (BMI). When positive, the results were tested by ANCOVA with the corresponding factor as covariate. In all the cases, a *p* value < 0.05 was considered statistically significant.

## Results

The different groups of controls, AD-free and AD-treated subjects at time of death, were comparable attending to gender ratio, age, postmortem delay (PMD), storage time, brain pH RNA integrity number (RIN), and blood ethanol concentrations (Additional file 1: Table S1). BMI was lower in MDD subjects than that in controls (Additional file 1: Table S1). Age at death significantly correlated only with Hsp70 expression (*r* = 0.54, *p* = 0.004). This demographic factor did not influence results due to the matched paired design. BMI showed inverse correlation with p-p38 (*r* = − 0.35, *p* = 0.007), ratio p-p38/total p38 (*r* = − 0.36, *p* = 0.005), and Keap-1 (*r* = − 0.28, *p* = 0.04) expression and positive correlation with NF-κB p65 expression (*r* = 0.31, *p* = 0.04). There was also a positive correlation between PMD and Hsp60 expression (*r* = 0.28, *p* = 0.03). Ethanol concentrations displayed significant correlation (*r* = 0.43, *p* = 0.003) with S100A10 (p11). Other drugs than antidepressants did not show influence on inflammatory and antioxidant parameters.

### Effects of MDD and antidepressant treatment on the expression of TLR-4 and its endogenous ligands Hsp60 and Hsp70

**Fig. 1:**
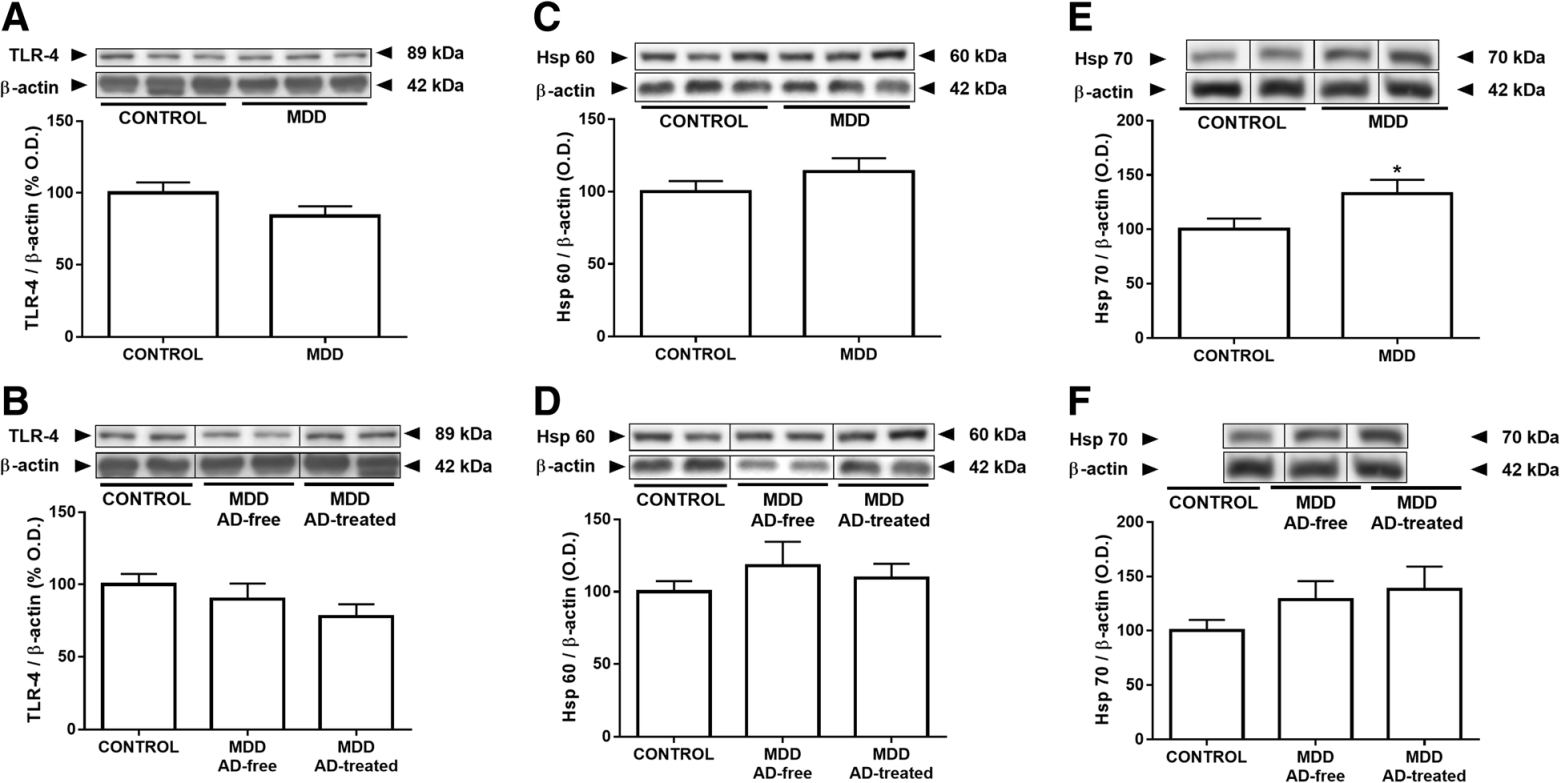
Effects of MDD and antidepressant treatment on the expression of TLR-4 and its endogenous ligands Hsp60 and Hsp70. There were no differences in the protein expression of TLR-4 among controls and subjects with MDD (**a**, **b**). There were no statistical differences between groups in the protein expression values of Hsp60 (**c**, **d**). Subjects with MDD showed a significant 33% increase in the Hsp70 protein expression compared with controls (**e**). The statistical significance disappeared when the AD-free at death and the AD-treated groups were split, but both groups maintained a trend to show higher values than control group (**f**). The densitometric data of the band of interest were normalized by *beta*-actin. Some blots were cropped (black lines) for improving the clarity and conciseness of the presentation. Data are means ± SEM; *p < 0.05 vs. control; unpaired two-tailed *t* test was performed when comparing controls vs MDD. One-way ANOVA with a Fisher’s LSD post hoc test was used to compare between controls and AD-free and AD-treated MDD groups. See the “Chemicals and statistical analyses” section for more details

TLR-4 expression analyses revealed no significant differences between controls and subjects with MDD (Fig. 1a, b).

There were no statistical differences either when the protein expression values of heat shock protein Hsp60 were analyzed (Fig. 1c, d). The results would not change when controlled for PMD effect. Conversely, subjects with MDD in bulk showed a significant 33% increase in the Hsp70 protein expression compared with controls (Fig. 1e). When the AD-free and the AD-treated at time of death groups were split, but both groups maintained a trend to show higher values than control group however without statistical significance (Fig. 1f).

### Effects of MDD and antidepressant treatment on the expression of MAPKs and the MAPK activity regulator DUSP2

**Fig. 2:**
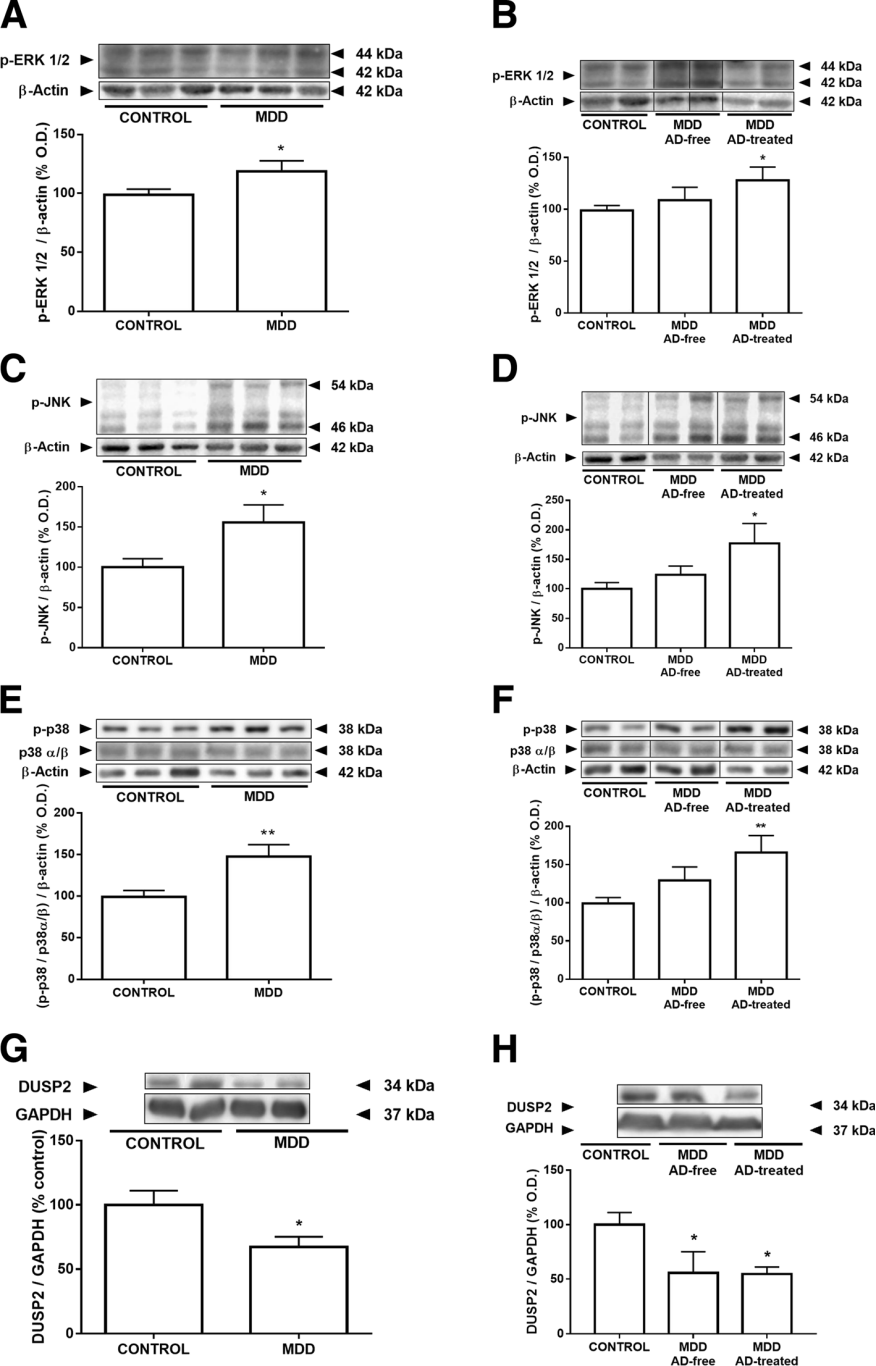
Effects of MDD and antidepressant treatment on the expression of MAPKs and on the expression of the MAPK activity regulator DUSP2. When considering the phosphorylated forms of the proteins, p-ERK1/2, p-JNK, and the ratio between p-p38/total p38 were clearly higher in MDD than in controls (**a**, **c**, **e**). The expression values were selectively increased in AD-treated subjects (**b**, **d**, **f**). Analysis of covariance controlling for BMI did not modify p-p38/total p38 ratio results. DUSP2 expression was decreased in MDD compared with matched controls (**g**), and this statistical difference appeared both in the subjects AD-free at time of death and in AD-treated subjects when compared with controls (**h**). The densitometric data of the band of interest were normalized by *beta*-actin. Some blots were cropped (black lines) for improving the clarity and conciseness of the presentation. Data are means ± SEM; *p < 0.05, **p < 0.01 vs. control; unpaired two-tailed *t* test was performed when comparing controls vs MDD. One-way ANOVA with a Fisher’s LSD post hoc test was used to compare between controls and AD-free and AD-treated MDD groups. See the “Chemicals and statistical analyses” section for more details

Subjects with MDD showed an increased expression of the activated (phosphorylated) forms of the extracellular signal-regulated kinases (ERK)1/2 (+ 22%, *t* = 2.293, *p* = 0.03) and the c-Jun N-terminal kinases (JNK) (+ 56%, *t* = 2.468, *p* = 0.02) but not the p38 MAPK (+ 19%, *t* = 1.317, *p* = 0.19) compared with matched controls (Fig. 2a, c). The activated forms of ERK1/2 and JNK increased in the AD-treated group when compared with the controls. Although phospho-p38 inversely correlates with BMI, ANCOVA demonstrated that controlling for this factor did not influence the results. When considering the ratio between the phosphorylated and the total forms of the proteins, the increase for p-ERK1/2/total ERK1/2 did not reach statistical significance whereas p-JNK/total JNK and p-p38/total p38 were clearly higher in MDD than in controls (Fig. 2e). The phosphorylated forms and ratios for p-ERK1/2/total ERK1/2, p-JNK/total JNK, and p-p38/total p38 were selectively increased in AD-treated subjects although did not reach statistical significance in the case of p-ERK1/2/total ERK1/2 ratio (Fig. 2b, d, f). ANCOVA controlling for BMI did not modify p-p38/total p38 ratio results.

Dual-specificity phosphatase 2 (DUSP2) regulates MAPK activity. DUSP2 expression decreased by 45% in MDD compared with matched controls (Fig. 2g). The statistical difference was retained both in the AD-free at time of death and the AD-treated groups when compared with controls (Fig. 2h).

### Effects of MDD and antidepressant treatment on the antioxidant nuclear factor (erythroid 2-derived)-like 2 (Nrf2) pathway

**Fig. 3:**
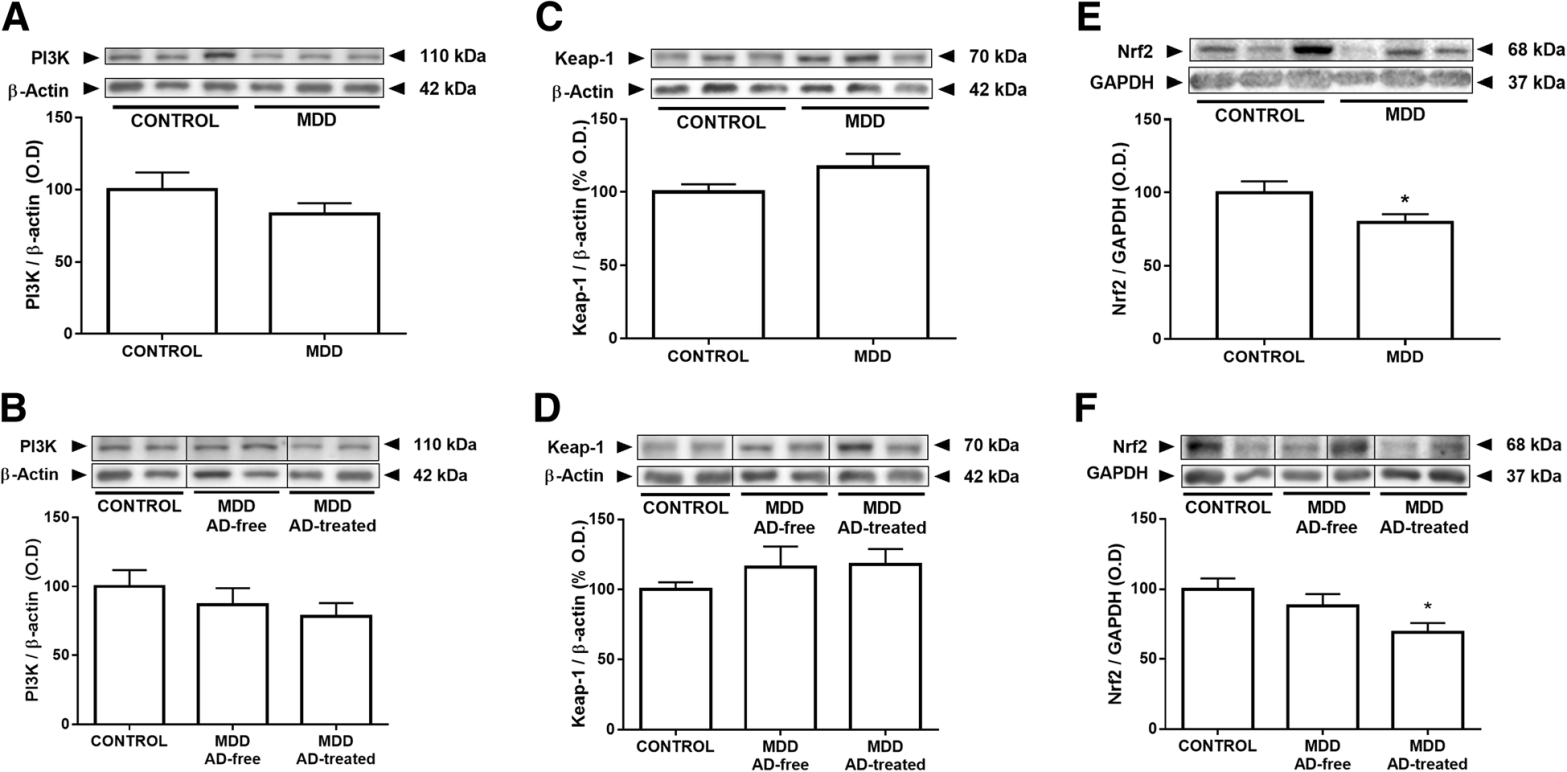
Effects of MDD and antidepressant treatment on the antioxidant nuclear factor (erythroid 2-derived)-like 2 (Nrf2) pathway. Subjects with MDD presented a non-significant trend to decrease (− 18%) the Nrf2 activator PI3K expression when compared with controls, being the decrease in the expression more pronounced in the AD-treated group (**a**, **b**). Subjects AD-free and AD-treated at death showed a non-significant trend towards a 16–18% increase in the expression of the cytoplasmatic inhibitor of Nrf2 Keap-1 protein when compared with controls (**c**, **d**). Subjects with MDD showed a significant decreased nuclear expression of Nrf2 (**e**). AD-treated group showed a decreased nuclear expression of Nrf2 compared with controls (**f**). The densitometric data of the band of interest were normalized by *beta*-actin or GAPDH for the cytoplasmic or nuclear fractions, respectively. Some blots were cropped (black lines) for improving the clarity and conciseness of the presentation. Data are means ± SEM; *p < 0.05 vs. control; unpaired two-tailed *t* test was performed when comparing controls vs MDD. One-way ANOVA with a Fisher’s LSD post hoc test was used to compare between controls and AD-free and AD-treated MDD groups. See the “Chemicals and statistical analyses” section for more details

The PI3K (phosphoinositide 3-kinase) is an activator of the antioxidant transcription factor Nrf2. Subjects with MDD presented an unaltered PI3K expression when compared with controls (Fig. 3a, b).

The Keap-1 (Kelch-like ECH-associated protein 1) is the cytoplasmic inhibitor of Nrf2. Subjects AD-free and AD-treated at time of death showed a non-significant increase in the expression of Keap-1 when compared with controls (Fig. 3c, d). Results of Keap-1 were not modified when controlling for BMI.

Subjects with MDD showed a 21% decrease of nuclear expression of Nrf2 (Fig. 3e). Statistical significance in the analysis of variance was observed only in the AD-treated group, which showed a 31% decrease of nuclear expression of Nrf2 compared with controls (Fig. 3f).

### Effects of MDD and antidepressant treatment on the expression of S100A10 (p11)

**Fig. 4:**
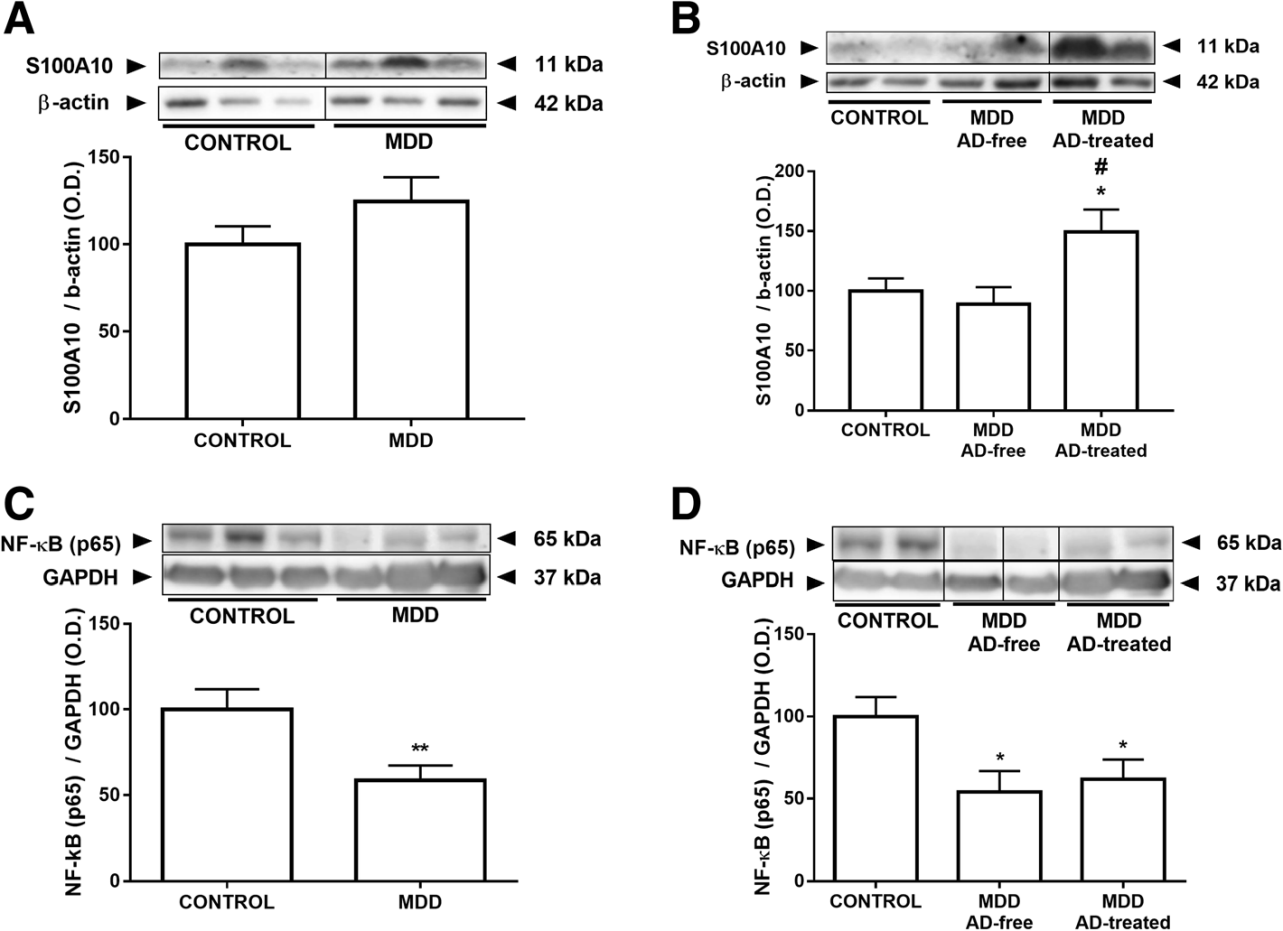
Effects of MDD and AD treatment on the expression of S100A10 (p11) and NF-κB. There were no significant differences in the p11 expression between control and MDD groups (**a**). p11 was elevated in the AD-treated at death group compared with the AD-free at death and the control groups (**b**). The p65 subunit of the transcription factor NF-κB expression was decreased in patients with MDD compared with controls (**c**) and when AD-free and AD-treated at death groups were compared with matched controls (**d**). The densitometric data of the band of interest were normalized by *beta*-actin or GAPDH for the cytoplasmatic or nuclear fractions, respectively. Some blots were cropped (black lines) for improving the clarity and conciseness of the presentation. Data are means ± SEM; *p < 0.05, **p < 0.01 vs. control; ^#^*p* < 0.05 vs. AD-free; unpaired two-tailed *t* test was performed when comparing controls vs MDD. One-way ANOVA with a Fisher’s LSD post hoc test was used to compare between controls and AD-free and AD-treated MDD groups. See the “Chemicals and statistical analyses” section for more details

The S100A10 (also known as p11) is a multifactorial protein linked with the transport of neurotransmitters and depression. There were no significant differences between the control and the MDD groups (Fig. 4a). However, when considering MDD subjects according to antidepressant treatment, p11 was elevated in the AD-treated group compared with the AD-free at time of death and the control groups (Fig. 4b). ANCOVA controlling for ethanol concentrations, maintained the significance of p11 increment in MDD.

### Effects of MDD and antidepressant treatment on the expression of NF-κB

The p65 subunit of the transcription factor NF-κB, which controls the transcription of many acute-phase proteins and inflammatory genes [29], was analyzed, showing decreased expression in subjects with MDD (Fig. 4c), both in AD-free (− 46%) and AD-treated (− 38%) groups at time of death (Fig. 4d). When results were controlled for BMI of the subjects, which has well-known effects on neuroinflammation [30], ANCOVA analysis failed to show statistical significance (*p* = 0.09).

## Discussion

The present work points to an alteration of inflammatory and oxidative/nitrosative pathways in the brain of subjects with MDD. Different cytoplasmic and nuclear factors involved in neuroinflammatory processes such as constituents of innate immunity response, elements of the antioxidant Nrf2 pathway, and transcription factors were assessed. Data indicate that the overexpression of proinflammatory MAPK pathways is associated with AD treatments, something partially described in in vitro and in vivo experimental settings [31–34] and in postmortem brain of subjects with MDD [28].

In the CNS, the innate immunity receptor TLR-4 is expressed in microglia, neurones, astroglia, oligodendroglia, and cerebral vascular endothelium [35]. Altered TLR-4 signaling has been described in peripheral blood cells and the brain of patients with schizophrenia, bipolar disease, and depression [10]; in postmortem brain of subjects with schizophrenia [27, 36]; and in stress animal models [10]. There are two predominant sources of TLR-4 activation: (i) altered gut microbiota [37] and the subsequent dysbiosis through increased intestinal barrier permeability and bacterial translocation present in patients with MDD [38, 39], and (ii) damage-associated molecular patterns (DAMPs) released by non-infective tissue injuries [40]. Among these DAMPs, the heat shock protein Hsp60 and the Hsp70 are well-known stimulators of the TLR-4 [16]. The present results indicate that TLR-4 expression was not noticeably induced in MDD subjects, at least at the moment when the samples were taken. These results also show that the treatment with antidepressants (AD-treated group) tends to decrease TLR-4 expression in the brain. Increased mRNA expression of TLR-4 and later reduction below control levels after AD treatment have been recently described in peripheral blood cells of patients with MDD [41]. In the dorsolateral prefrontal cortex of depressed subjects, TLR-4 increased mRNA expression has been previously found together with an unaltered TLR-4 protein expression in non-suicide victims and elevation in suicide victims [42]. In this previous study, although the influence of AD treatment was considered, a controlled design for this purpose was not included which prevents from drawing definitive conclusions. Consistent with the hypothesis of a DAMP activation of TLR-4 pathways in MDD, evidence of enhanced Hsp70 protein expression is here provided. No differences in Hsp70 expression are observed between AD-free at time of death and AD-treated subjects, suggesting that elevation of the endogenous ligand Hsp70 is rather associated to the disorder and that downregulation of the TLR-4 receptor after AD treatment represents the compensatory effect against the hyperactive pathway. An equivalent observation has been found for the Hsp70 family member GRP78 in postmortem brain of depressed suicide victims [43]. Whether increased Hsp70 expression represents a dysfunction originated in the periphery or a primary CNS impairment remains to be elucidated.

The MAPKs are downstream elements activated after TLR-4 stimulation [17, 44]. The MAPK pathways are implicated in a wide range of signaling cascades wherein various extracellular stimuli induce inflammation. It has been shown that MAPKs can play as well an important role in the regulation of the NF-κB pathway [45]. Moreover, proinflammatory cytokines can increase, through activation of signaling pathways that include MAPKs, the expression and function of monoamine transporters [46, 47], the most important target for antidepressant drugs. In this context, genes related to MAPK pathways seem to be upregulated by chronic antidepressant treatment [33]. Data suggest increased expression of the activated forms of ERK1/2, JNK, and p38 proteins in MDD. When the MDD group is evaluated considering the presence of AD drugs, the elevation is mainly observed in AD-treated subjects. Thus, the presence of AD treatment might be associated with an overactivation of MAPK pathways.

Changes in basal ERK activation produce structural changes in neurones responsible for phenotypes of mood disorders [48]. ERK has been related to the regulation of gene expression and protein synthesis, shifts in the cellular structure and metabolism, cell differentiation and apoptosis. All these regulatory effects might play a role in the impairments of neural plasticity, neurogenesis, and cellular resilience described in the brain of subjects with depression [49]. Furthermore, the effectiveness of drugs such as mood stabilizers and antidepressants partially depends on their capacity to stimulate ERK in the CNS [49, 50]. In postmortem frontal cortex of suicide victims with MDD, mainly in AD-free conditions, a decreased expression and activation of total ERK1/2 has been previously described [51]. Another study in postmortem brain has found unaltered total ERK1/2 protein expression with decrease of active p-ERK1/2 forms that are normalized under AD treatment [28]. These data are quite consistent with results in rats exposed to stress where chronic fluoxetine reversed the lower p-ERK1/2 protein expression in the hippocampus and frontal cortex [32]. Accordingly, in the present study, subjects AD-free at time of death show a non-significant dropping tendency (− 26%) of total ERK1/2 expression when compared to controls whereas only a 7% reduction is obtained in AD-treated (data not shown). On the other hand, the expression of p-ERK1/2 (activated) form and p-ERK1/2/total ERK1/2 ratio is markedly increased in MDD subjects under AD treatment. Thus, AD treatment seems to promote expression of activated ERK forms, possibly in an attempt to recover the plasticity and counteract the negative cellular effects associated with the reduced expression of ERK observed in the disorder.

An increased expression of the activated forms of JNK and p38 in subjects with MDD is shown here. Importantly, their expression follows the same pattern as the expression of ERK, being higher in the AD-treated group. Both p38 and JNK have been linked to the pathophysiology of depression [52]. Specifically, p38 has been related to MDD because of its proinflammatory actions and its stimulatory activity on the expression and function of the serotonin transporter [2]. JNK plays a critical role in the regulation of T lymphocyte differentiation and cytokine production [52]. Inhibition of the JNK pathway in animal models of depression [53] and activation of p38-dependent pathways by the antidepressant amitriptyline [54] have been previously described. Further research is needed to evaluate the role of these MAPKs in MDD and their modulation by AD drugs.

Dual-specificity phosphatases (DUSPs) are a heterogeneous group of protein phosphatases that regulate activity of MAPKs through dephosphorylation (deactivation) and serve to control the MAPK subcellular localization [55]. DUSP2 displays substrate specificity for ERK, p38, and JNK. In fact, preclinical in vivo studies revealed that DUSP2 is a negative regulator of JNK activity and positive regulator of immune responses via cross-talk between JNK and ERK [56]. The present results show a decrease in DUSP2 expression in the MDD group, both in subjects AD-free and AD-treated at time of death. This result could explain, at least partially, the trend towards enhanced MAPK expression in MDD.

Nrf2 is a main cellular defense pathway activated by oxidative stress, leading to production of target antioxidants [57]. A role for the Nrf2 pathway in experimental models of depression has been described [58, 59]. Thus, Nrf2 null mice are more vulnerable to develop depression-like phenotypes [60]. Indeed, Nrf2 has been proposed as a potential therapeutic target worth to consider in future studies [61]. Our data indicate a decrease of the Nrf2 pathway in subjects with MDD. This decrease would be in agreement with the proposed role of Nrf2 in the pathophysiology of depression, being involved in the enhanced oxidative/nitrosative stress described in this disorder [62]. Antidepressant treatment affected the nuclear expression of Nrf2. Previous preclinical studies showed that antidepressants modulate this pathway and that this effect differs depending of the CNS region analyzed [59]. Moreover, there are results in human monocytic U-937 cells, suggesting that the effects of AD on the expression of antioxidant enzymes are dependent on the duration of the treatment and even can have pro-oxidant consequences [63]. Here, AD treatments do not reverse the trend to reduction observed in AD-free at time of death subjects and even could be negatively affecting the Nrf2 protective pathway. The possibility that both groups of subjects could represent a population with resistance to AD should bear in mind due to the presence of suicide as the main cause of the death. The downward trend in the expression of the Nrf2 activator PI3K and increased expression of the Nrf2 inhibitor Keap-1 both in AD-free and AD-treated populations at time of death is congruent with the nuclear reduction of this transcription factor.

The calcium-binding protein p11 is linked with the transport of neurotransmitters and depression and data from cell lines confirm that it is regulated by immunological parameters like TLR-4 [64]. Administration of proinflammatory cytokines stimulates the formation of p11 through unknown mechanisms [65]. Experimental data indicate that p11 knockout mice display a depressive-like phenotype whereas p11 overexpressing mice display antidepressant-like responses [66]. Moreover, p11 is upregulated in the CNS after chronic antidepressant treatments. Once upregulated, p11 increases the expression of 5-HT receptors on the plasma membrane, providing an enhancer mechanism for antidepressant actions [67]. Our results show a specific augmentation of p11 protein expression in the AD-treated group compared with the AD-free group at time of death and controls. The data agree with prior results in animal models of depression and humans suffering with MDD where decreased p11 expression was reported [66]. However, it is the first evidence showing that AD treatment may result in increased p11 protein expression reported for MMD subjects.

NF-κB is a nuclear factor that controls the transcription of many acute-phase proteins and inflammatory genes, being one of the early events in the stress-induced inflammatory response in CNS [26]. Although hyperactive NF-κB in peripheral cells has been considered a fingerprint of stress and mood disorders, in animals under chronic mild stress conditions, small reductions of NF-κB p65 mRNA and protein expression and activity have been described [26]. The present study suggests a similar tendency in the human brain. However, control subjects exhibited a higher BMI, a physical parameter that displayed positive relationship with NF-κB expression. Therefore, larger studies of controlled populations for BMI are necessary to establish definitive conclusions on the status of NF-κB in the brain of subjects with MDD. Besides, NF-κB is not only a main regulator of the inflammatory response but is also an essential regulatory element of cell survival, controlling key processes such as neuroprotection, neuronal transmission, and plasticity [68]. Once again, more studies are warranted in order to fully understand the role of NF-κB in this particular setting.

Suicide represents a special cause of death that may act as a confounding factor in studies using postmortem brain samples from subjects with psychiatric disorders. Since suicide is highly prevalent in depressive disorders, the neurobiology underlying suicide might mask some results in MDD studies. Therefore, the assumption that description of inflammation pathways altered in the brain of depressed suicide victims can automatically be extended to depressed non-suicide victims is potentially faulty. In fact, case points of differences between suicide and non-suicide depressed subjects for TLR-4 [42] and microglial density [69] have been published. Moreover, MDD subjects died by suicide and under confirmed antidepressant treatment, as herein presented, should be considered more representative of resistant than standard depression. Although suicide was the cause of death for an important number of MDD subjects in the present study, opposite results for TLR-4 and NF-κB expression have been recently described in a schizophrenia population mainly dead by suicide [27]. Therefore, at least some of the changes herein observed in the postmortem brain of MDD subjects should be associated to depression and antidepressant treatment rather than to suicide behavior. About 4% of depressed individuals die by suicide, being the risk greater in males [70]. Although depression is more prevalent in female, this factor could explain the higher presence of male subjects in the present study.

**Fig. 5:**
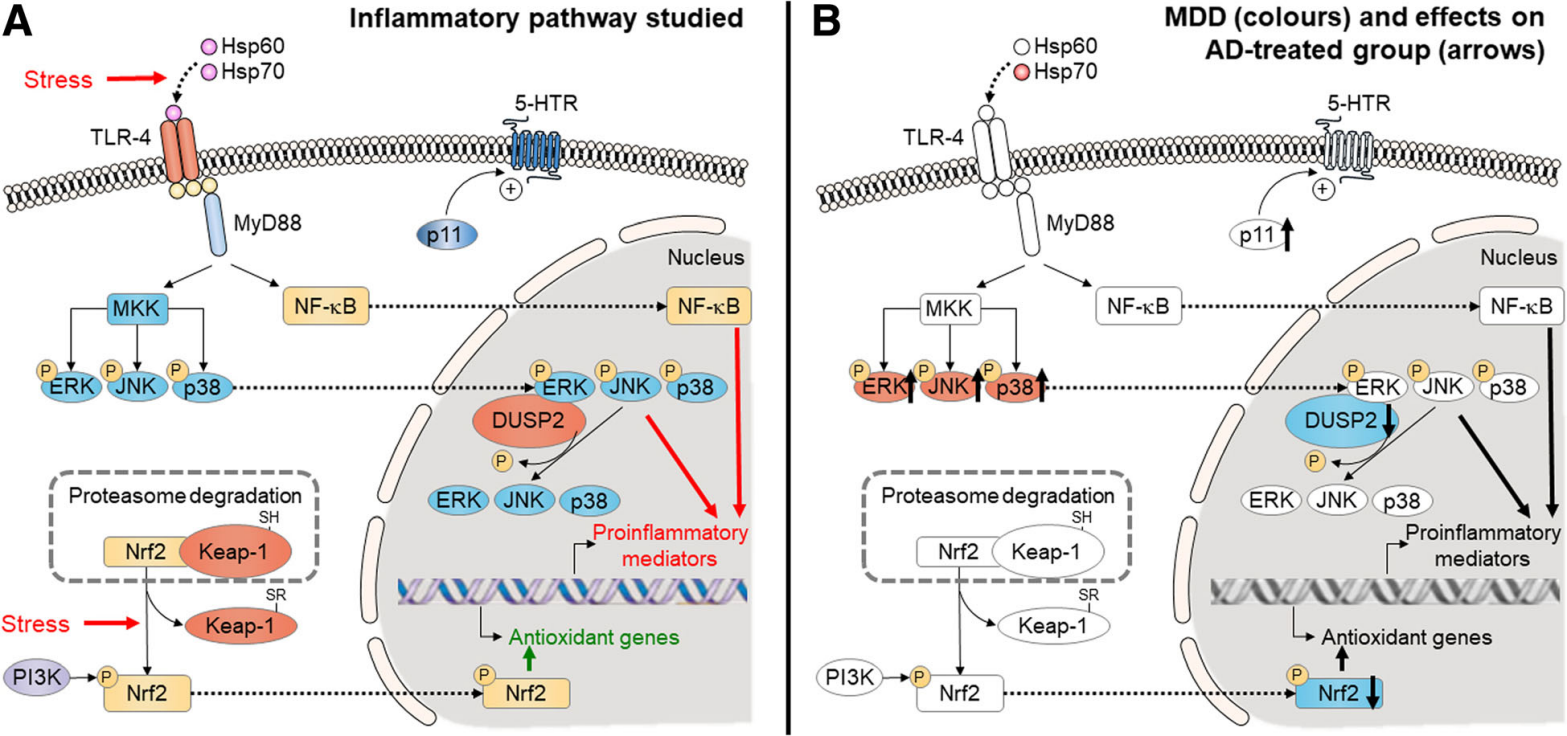
Schematic representation of the pathways being affected by MDD and by treatments. Inflammatory pathway studied (**a**) and results obtained (**b**). In **b**, the colors show the statistically significant changes in major depressive disorder (MDD) vs. control group (red = upregulation, blue = downregulation). The arrows indicate the effects on the MDD group treated with antidepressants at time of death (AD-treated group). TLR Toll-like receptor, Hsp Heat shock protein, MyD88 myeloid differentiation primary response 88, MKK mitogen-activated protein kinase kinase, ERK extracellular signal-regulated kinase, JNK c-Jun N-terminal kinase, p38 protein 38, NF-κB nuclear factor kappa B, 5-HTR serotonin receptor, p11 S100 calcium-binding protein A10 (S100A10), DUSP2 dual-specificity phosphatase 2, Nrf2 nuclear factor (erythroid 2-derived)-like 2, Keap-1 Kelch-like ECH-associated protein 1, PI3K phosphoinositide 3-kinase, P phosphate

The fact that selection of MDD subjects was performed based on clinical records of subjects could be interpreted as a study limitation. In fact, the use of *antemortem* diagnosis versus *postmortem* retrospective methodologies based on psychological autopsy is still a matter of controversy [71]. The psychopathological condition of MDD and control subjects at time of death represents also a limitation of this type of studies performed in outpatients who die by sudden and unexpected causes. On the other hand, although control subjects did not show clinical records of neurological or psychiatric disorders, including drug abuse, the possibility of presence of potential non-diagnosed subjects among controls is not completely ruled out. For a better interpretation of the alterations caused by MDD and the actions of antidepressant treatments see Fig. 5.

## Conclusions

Our results show an increase in Hsp70, an endogenous ligand of the TLR-4 and in the products of the TLR-4 activation such as MAPKs in postmortem dorsolateral prefrontal cortex of subjects with MDD. An important effect to consider is the influence of the pharmacological treatment with AD on MAPK expression. Further research focused on the mechanisms that contribute to this modulation is essential and could help developing new treatment strategies for MDD.

Results also show for the first time in the brain of MDD subjects that the disorder is affecting the antioxidant Nrf2 pathway. In particular, a decrease in the antioxidant factor Nrf2 is observed. Complementary, AD treatment induces an increase of p11 that could be boosting the expression of 5-HT receptors.

The data seem to point out to the presence of an altered innate immune response in the brain of subjects with MDD, in whom the TLR4 pathway could be one of the main elements influenced. However, Hsp70 also binds to other TLRs as well as other receptors and therefore additional studies are required to explicitly confirm TLR-4 involvement. Similarly, further research is necessary to ascertain the condition of the innate immune system in the MDD and attending especially to the potential confounding factor of antidepressant treatment.

## Additional file

Additional file 1: Supplemental Material. (DOC 71 kb)

## Abbreviations

AD: Antidepressants
BMI: Body mass index
CNS: Central nervous system
DAMPs: Damage-associated molecular patterns
DUSP2: Dual-specificity phosphatase 2
ERK1/2: Extracellular signal-regulated kinases 1/2
Hsp: Heat shock protein
JNK: c-Jun N-terminal kinases
Keap-1: Kelch-like ECH-associated protein 1
LPS: Lipopolysaccharide
MAPKs: Mitogen-activated protein kinases
MDD: Major depressive disorder
NF-κB: Nuclear factor *kappa* B
Nrf2: Nuclear factor (erythroid 2-derived)-like 2
PI3K: Phosphoinositide 3-kinase
PMD: Postmortem delay
RIN: RNA integrity number
TLR: Toll-like receptor
