# Supplementary material for: Intracellular inflammatory and antioxidant pathways in postmortem frontal cortex of subjects with major depression: effect of antidepressants
Source: J Neuroinflammation. 2018 Sep 4;15:251. doi: 10.1186/s12974-018-1294-2 (PMC6122627; doi:10.1186/s12974-018-1294-2)
Supplement: Supplementary file 1 — Supplemental Material. (DOC 71 kb) [file 12974_2018_1294_MOESM1_ESM.doc]

**Supplemental Material**

**Postmortem human brain samples:**

Human brain samples were obtained at autopsy in the Basque Institute of Legal Medicine, Bilbao, Spain in compliance with research policies and ethical committees for postmortem brain studies. After a retrospective search for *antemortem* medical information, 30 subjects diagnosed with MDD (DSM-IV, DSM-IV-R or CIE-10 criteria), were matched for gender and age (±4 years) to 30 control subjects in a paired design. MDD subjects with drug abuse or dependence criteria were excluded. The criteria for selecting control subjects were absence of neuropsychiatric disorders and absence of drug abuse. Sudden and unexpected deaths were preferentially selected in order to avoid possible confounding agonal influences. Blood from all the subjects was screened to determine the presence of antidepressants, other drugs and ethanol at the time of death. Since non-compliance is frequently observed, the absence or presence of antidepressant drugs was defined according to the toxicological screening at the time of death: Thus, MDD subjects were divided into two groups: antidepressant-free at time of death (AD-free; n=15) and antidepressant-treated at time of death (AD-treated; n=15).

Specimens of dorsolateral prefrontal cortex (Brodmann’s area 9) were dissected at autopsy (0.5–1 g tissue) following standard procedures (Rajkowska and Goldman-Rakic, 1995). They were immediately stored at -80°C until assayed. The quality indexes brain pH and RNA integrity number (RIN) were measured as previously reported (Garcia-Sevilla et al., 2010 Muguruza et al., 2013). Other confounding factors as the interval between death and autopsy (postmortem delay, PMD), the storage period and the influence of blood ethanol concentrations were also calculated. The values for these parameters are shown in Table S1.

## **Preparation of nuclear and cytosolic extracts**

Briefly, the brain tissue was homogenized in 600 μL buffer [10 mmol/L N-2-hydroxyethylpiperazine-N-2-ethanesulfonic acid (pH 7.9); 1 mmol/L EDTA, 1 mmol/L EGTA, 10 mmol/L KCl, 1 mmol/L dithiothreitol, 0.5 mmol/L phenylmethylsulfonyl fluoride, 0.1 mg/mL aprotinin, 1 mg/mL leupeptin, 1 mg/mL Na-p-tosyll-lysine-chloromethyl ketone, 5 mmol/L NaF, 1 mmol/L NaVO_4_, 0.5 mol/L sucrose, and 10 mmol/L Na_2_MoO_4_]. After 15 min, Nonidet P-40 (Roche, Mannheim, Germany) was added to reach a 0.5% concentration. The tubes were gently vortexed for 15 sec, and nuclei were collected by centrifugation at 5000g for 5 min. Supernatants were considered as the cytosolic fraction. The pellets were re-suspended in 100 μL buffer supplemented with 20% glycerol and 0.4 mol/L KCl and gently shaken for 30 min at 4ºC. Nuclear protein extracts were obtained by centrifugation at 13,000g for 5 min, and aliquots of the supernatant were stored at -80ºC. All steps of the fractionation were carried out at 4ºC.

**Western blot analysis**

The expression levels of TLR-4, Hsp60, Hsp70, phospho-ERK 1/2, phospho-JNK, phospho-p38, p38 α/β, PI3K, Keap-1 and S100A10 (p11) in cytosolic extracts and the expression levels of PAC-1 (DUSP-2), Nrf-2 and p65 (NF-κB subunit) in nuclear extracts from brain samples were analyzed through Western blot.

Protein levels were adjusted and homogenates mixed with Laemmli sample buffer (BioRad, Hercules, CA, USA) and 15 μL (1 mg/mL) were loaded and the proteins size-separated in 10% SDS-polyacrylamide gel electrophoresis (90 V). In the case of nuclear factors, analyses were carried out in nuclear extracts. All the procedures were performed at 4ºC.

After the gel electrophoresis the membranes were blocked in 30ml Tris-buffered saline containing 0.1% Tween 20 and 5% skim milk/BSA, then the membranes were incubated with specific primary antibodies against ^Thr202/Tyr204^phospho-ERK and ^Thr183/Tyr185^phospho-SAPK/JNK (Cell Signaling, 1:1000); ^Thr180/Tyr182^phospho-p38, p38 α/β, Hsp70, Nrf2 and p65 (Santa Cruz Biotechnology, 1:1000); S100A10 (Abcam, 1:500). After washing with a TBS-Tween solution the membranes were incubated with the respective horseradish peroxidase-conjugated secondary antibodies for 90 min at room temperature and revealed by ECL^TM^-kit following manufacturer’s instructions (Amersham Ibérica, Spain).

Blots were imaged using an Odyssey® Fc System (Li-COR Biosciences) and quantified by densitometry (NIH ImageJ® software). All densitometries are expressed in arbitrary units of optical density (O.D.) as percentage from control. Several exposition times were analyzed to ensure the linearity of the band intensities. The loading controls were β-actin (Sigma A5441) for the cytosolic fraction and GAPDH (Sigma G8795) for the nuclear fraction (representative blots shown in the respective figures). In some cases blots have been cropped (indicated with black lines) for improving the clarity and conciseness of the presentation.

**References:**

Garcia-Sevilla JA, Alvaro-Bartolome M, Diez-Alarcia R, Ramos-Miguel A, Puigdemont D, Perez V, Alvarez E, Meana JJ : Reduced platelet G protein-coupled receptor kinase 2 in major depressive disorder: antidepressant treatment-induced upregulation of GRK2 protein discriminates between responder and non-responder patients. Eur. Neuropsychopharmacol. 2010, 20: 721-730.

Muguruza C, Moreno JL, Umali A, Callado LF, Meana JJ, González-Maeso J: Dysregulated 5-HT2A receptor binding in postmortem frontal cortex of schizophrenic subjects. Eur. Neuropsychopharmacol. 2013, 23: 852-864.

Rajkowska G, Goldman-Rakic PS: Cytoarchitectonic definition of prefrontal areas in the normal human cortex: I. Remapping of areas 9 and 46 using quantitative criteria. Cereb. Cortex 1995, 5: 307-322.

**Table S1.** Demographic characteristics, postmortem delay (PMD), storage time, body mass index (BMI), RNA integrity number (RIN), cause of death/mechanism, brain pH and toxicological analysis of the subjects with major depressive disorder (MDD) and matched control subjects (C).

|  | **Antidepressant-free MDD subjects (n=15) and matched controls (n=15)** | | | | | | | | | | |
| --- | --- | --- | --- | --- | --- | --- | --- | --- | --- | --- | --- |
| **Case/Control** | | **Gender** | **Age** | **PMD** | **Storage time** | **BMI** | **RIN** | **Cause of death/Mechanism** | **Brain** | **Antidepressants** | **Other drugs in** |
| **(MDD/C)** | | **(F/M)** | **(years)** | **(hours)** | **(months)** |  |  |  | **pH** | **in blood** | **blood** |
| MDD1  C1  MDD 2  C2  MDD 3  C3  MDD 4  C4  MDD 5  C5  MDD 6  C6  MDD 7  C7  MDD 8  C8  MDD 9  C9  MDD 10  C10  MDD 11  C11  MDD 12  C12  MDD 13  C13  MDD 14  C14  MDD 15  C15 | | M  M  M  M  M  M  M  M  F  F  M  M  M  M  F  F  M  M  M  M  F  F  M  M  M  M  M  M  M  M | 30  30  31  23  43  42  35  33  26  28  48  45  59  58  52  50  48  47  56  54  39  38  50  51  34  36  42  41  32  31 | 4  11  23  17  15  27  13  4  26  55  32  30  31  20  18  11  15  15  22  24  17  22  24  18  18  18  16  14  16  13 | 132  134  82  105  100  198  153  111  235  237  239  238  195  235  74  79  106  56  69  63  70  58  68  51  150  152  49  73  97  111 | 21  25  23  28  31  24  23  31  19  23  24  23  18  25  26  24  30  30  23  27  24  22  23  30  27  30  23  24  25  35 | 8.0  8.0  9.0  7.1  N/A  N/A  N/A  9.1  N/A  N/A  N/A  6.3  N/A  6.2  8.1  8.2  7.9  7.4  N/A  N/A  7.2  7.6  N/A  N/A  9.0  9.2  N/A  N/A  7.1  8.2 | Suicide/Jumping  Accident/Electric shock  Suicide/Hanging  Accident/Electric shock  Suicide/Train  Accident/MVA  Suicide/Hanging  Accident/MVA  Suicide/Jumping  Accident/MVA  Suicide/Hanging  Accident/MVA  Suicide/Hanging  Accident/Falling from a height  Suicide/Jumping  Natural/CRF  Natural/CRF  Accident/MVA  Suicide/GSW  Accident/MVA  Natural/CRF  Accident/MVA  Suicide/GSW  Accident/MVA  Suicide/Jumping  Accident/Crushing  Suicide/Drowned  Natural/Heart attack  Suicide/Jumping  Accident/MVA | 7.09  7.10  6.33  N/A  N/A  N/A  N/A  N/A  N/A  N/A  N/A  6.82  N/A  6.74  6.2  6.21  N/A  6.15  6.19  6.33  6.35  6.44  6.8  6.2  N/A  N/A  6.3  6.81  6.33  N/A |  | THC  ETH (1.02g/l), BZD  ETH (3.09g/l)  ETH (0.99g/l)  ETH (3.20g/l)  ETH (1.55g/l)  ETH (0.60g/l)  ETH (1.42g/l)  BZD  ETH (1.69g/l)  AMP  AMP, THC  ETH (0.96g/l) |
| Group MDD | | 12M/3F | 42±3 | 19±2 | 121±16 | 24±1 | 8.0±0.3 |  | 6.5±0.1 | |  |
| Group C | | 12M/3F | 41±3 | 20±3 | 127±18 | 27±1 | 7.7±0.3 |  | 6.5±0.1 | |  |

|  | |  |  |  |  |  |  |  |  | |  |
| --- | --- | --- | --- | --- | --- | --- | --- | --- | --- | --- | --- |
|  | **Antidepressant-treated MDD subjects (n=15) and matched controls (n=15)** | | | | | | | | | | |
| **Case/Control** | | **Gender** | **Age** | **PMD** | **Storage time** | **BMI** | **RIN** | **Cause of death / Mechanism** | **Brain** | **Antidepressants** | **Other drugs in** |
| **(Sch/C)** | | **(F/M)** | **(years)** | **(hours)** | **(months)** |  |  |  | **pH** | **in blood** | **blood** |
| MDD 16  C16  MDD 17  C17  MDD 18  C18  MDD 19  C19  MDD 20  C20  MDD 21  C21  MDD 22  C22  MDD 23  C23  MDD 24  C24  MDD 25  C25  MDD 26  C26  MDD 27  C27  MDD 28  C28  MDD29  C29  MDD30  C30 | | M  M  M  M  M  M  F  F  M  M  M  M  M  M  F  F  F  F  M  M  F  F  M  M  M  M  M  M  F  F | 37  36  61  58  45  43  49  45  43  44  35  32  47  47  34  32  74  75  34  37  60  57  50  51  60  61  70  71  70  70 | 3  23  5  16  6  10  18  12  34  21  20  28  18  39  6  18  22  39  10  14  17  14  23  13  18  23  16  21  14  7 | 49  63  148  151  81  142  92  81  105  104  91  139  105  132  131  133  107  190  63  132  65  96  93  58  120  152  129  139  139  137 | 22  33  30  30  26  23  22  31  23  31  25  25  21  29  21  21  25  28  26  23  22  23  24  29  28  27  18  28  31  34 | 7.6  8.2  N/A  N/A  8.1  7.1  8.0  7.9  8.4  8.5  8.2  8.0  N/A  N/A  N/A  N/A  8  N/A  N/A  N/A  N/A  N/A  8.2  8.1  8.0  8.0  N/A  N/A  N/A  N/A | Suicide/Jumping  Accident/Falling from a height  Natural/CRF  Accident/MVA  Suicide/Jumping  Accident/MVA  Suicide/Drowned  Natural/Heart attack  Suicide/Hanging  Accident/MVA  Suicide/GSW  Accident/MVA  Suicide/Hanging  Accident/MVA  Suicide/Overdose  Accident/MVA  Suicide/Jumping  Accident/MVA  Suicide/Hanging  Accident/MVA  Suicide/Jumping  Natural/CRF  Suicide/GSW  Accident/MVA  Suicide/Hanging  Accident/MVA  Suicide/Hanging  Natural/CRF  Natural/CRF  Accident/MVA | 6.56  6.42  N/A  N/A  6.48  N/A  6.56  6.37  N/A  N/A  5.98  6.83  N/A  N/A  N/A  N/A  5.70  N/A  6.50  N/A  6.49  N/A  6.23  6.31  N/A  7  N/A  N/A  N/A  N/A | MIR, PAR  FLU  MIR  FLU  CIT  FLU  CIT  VEN, CIT  SER  CMI  VEN  VEN  FLU  MIR, VEN  CIT | BZD  ETH (0.77g/l)  BZD  ETH (2.50g/l)  ETH (0.51g/l)  BZD  BZD, COC, ETH (0.74g/l)  ETH (1.16g/l)  BZD, CLZ, TOP  ETH (1.21g/l)  TRA  ETH (2.20g/l)  ETH (1.04g/l)  ETH (2.13g/l)  BZD, OLZ  ETH (0.72g/l) |
| Group MDD | | 10M/5F | 51±4 | 15±2 | 101±8 | 25±1 | 8.0±0.1 |  | 6.3±0.1 | |  |
| Group C | | 10M/5F | 51±4 | 20±3 | 123±9 | 27±1 | 8.0±0.2 |  | 6.6±0.1 | |  |
|  | **AD-free and AD-treated MDD subjects (n=30) and matched controls (n=30)** | | | | | | | | | | |
| Group MDD | | 22M/8F | 46±2 | 17±1 | 111±9 | 24±1 | 8.1±0.1 |  | 6.4±0.1 | |  |
| Group C | | 22M/8F | 46±2 | 20±2 | 125±10 | 27±1 | 7.8±0.2 |  | 6.6±0.1 | |  |

**Table S1** *(cont.)*

Group values are means±SEM. F (female), M (male), CRF (cardio-respiratory failure), GSW (gunshot wound), MVA (motor vehicle accident), N/A (not available). Drugs in blood are coded as AMP (amphetamine), BZD (non-especified benzodiazepines or metabolites), CIT (citalopram), CLZ (clozapine), CMI (clomipramine), COC (cocaine), ETH (ethanol), FLU (fluoxetine), MIR (mirtazapine), OLZ (olanzapine), PAR (Paroxetine), SER (sertraline), THC (tetrahidrocannabinol), TOP (topiramate), TRA (tramadol), VEN (Venlafaxine).
